# Importance-Aware Resource Allocations for MIMO Semantic Communication

**DOI:** 10.3390/e27060605

**Published:** 2025-06-05

**Authors:** Yue Cao, Youlong Wu, Lixiang Lian, Meixia Tao

**Affiliations:** 1 School of Information Science and Technology, ShanghaiTech University, Shanghai 201210, China; caoyue@shanghaitech.edu.cn (Y.C.); lianlx@shanghaitech.edu.cn (L.L.); 2 Department of Electronic Engineering and the Cooperative Medianet Innovation Center (CMIC), Shanghai Jiao Tong University, Shanghai 200240, China; mxtao@sjtu.edu.cn

**Keywords:** semantic communication, separate source-channel coding (SSCC), attribution, MIMO

## Abstract

This study proposes a separate source-channel coding (SSCC) framework to address semantic communication challenges in MIMO systems, overcoming the limitations of joint source-channel coding (JSCC) in channel adaptation and model reusability. Traditional systems suffer from bit-level redundancy in 6G, while JSCC struggles with complex channel variations. Our solution decouples semantic processing from channel coding through a three-tier architecture: (1) Variational autoencoder (VAE)-based semantic encoder and decoder for source coding, (2) A communication-informed bottleneck attribution (CIBA) mechanism quantifying feature importance for learning tasks, and (3) An importance-aware resource allocation scheme aligning communication objectives with deep learning tasks. Systematic experiments validate CIBA’s effectiveness in deriving importance scores that bridge learning tasks and communication optimization. Comparisons of feature perturbation schemes confirm the necessity of importance-aware resource allocation, with the proposed allocation strategy outperforming conventional methods in task performance metrics. The SSCC design enhances model reusability while maintaining adaptability to diverse MIMO configurations. By integrating interpretable AI with resource management, this work establishes a foundation for SSCC semantic communication systems in resource-constrained environments, prioritizing semantic fidelity and task efficacy over bit-level redundancy. The methodology highlights the critical role of importance awareness in optimizing both communication efficiency and learning task performance.

## 1. Introduction

Traditional communication systems are designed following Shannon’s coding theory, aiming to transmit information bits as accurately as possible over noisy channels without considering the semantic information contained within the data. In fact, with the development of artificial intelligence (AI) technologies, practical application scenarios such as semantic communications [1] have demonstrated that accurate transmission of semantic-level information alone can achieve task objectives. In such cases, traditional communication systems’ pursuit of lossless bit-level transmission strategies would introduce significant information redundancy. Emerging 6G applications, including autonomous driving, remote robotics, meta-universe, smart healthcare, etc., involve massive data transmission demands driven by large-scale connected intelligent machines, while requiring ultra-low communication latency. It is foreseeable that in the 6G era, conventional communication systems will reach their limits in supporting future ubiquitous-connectivity and machine-intelligence services [2]. This urgency necessitates a fundamental shift in communication system design from traditional bit-level transmission to semantic or effectiveness-level considerations. As a novel communication paradigm, semantic communication focuses on information transmission at semantic or effectiveness levels, significantly reducing communication overhead through semantic extraction and transmission. Current research progress in semantic communication has demonstrated higher communication efficiency, improved reliability, and better compatibility with emerging AI applications compared to traditional systems, particularly in text [3], image [4,5], speech [6], and video [7] transmission scenarios.

The ultimate goal of semantic communication is to leverage intrinsic semantic information from sources to convey the sender’s intended meaning to receivers. Historically, the lack of a unified theoretical framework for representing semantics across different sources made semantic information difficult to represent and quantify. However, the powerful representation and generalization capabilities of deep neural networks (DNNs) have enabled semantic extraction. In the current research [8], semantic communication systems typically employ DNN-parameterized feature encoders and decoders to extract and decode semantic representations. Through end-to-end training with incorporated communication constraints, these systems achieve joint design of source-channel coding. The semantic encoder integrates source and channel coding functions, enabling extracted representations to preserve semantic information while maintaining excellent communication performance. This deep learning-based joint source-channel coding (JSCC) approach, implemented through neural network parameterization and end-to-end training, has been extensively studied and validated in SISO scenarios [9,10,11,12].

However, unlike the simple model structure in SISO scenarios, MIMO systems face more complex channel conditions. The high dimensionality of MIMO channel matrices leads to exponential growth in potential channel state combinations. End-to-end JSCC semantic communication networks require parameter learning based on task datasets and the entire distribution of wireless channels, resulting in substantial training overhead and unpredictable complexity [13]. Training end-to-end networks to adapt to diverse communication environments often proves impractical. Existing MIMO semantic communication systems, such as the speech-to-text system proposed in [14], employ JSCC but lack CSI adaptive design. The work in [15] utilized vision transformer models for semantic encoder and decoder with JSCC, training separate models for different Signal-to-Noise Ratio (SNR). Their results show optimal performance only when training and testing SNRs match. Although they propose a stochastic SNR training scheme for channel adaptation, this compromises performance compared to non-adaptive approaches. Parameters in JSCC models typically cannot be reused under different channel conditions, necessitating additional training resources and specialized designs for channel adaptation.

The fundamental issue in JSCC stems from semantic encoders being burdened with channel coding tasks, forcing semantic encoder and decoder to memorize complex MIMO channel conditions during training. By decoupling channel coding from source coding through separated design, we can eliminate channel dependence during training, reducing training complexity and overhead. However, a critical challenge emerges in separated designs: how to establish connections between learning tasks and communication objectives through channel coding schemes. In limited related work, Cai et al. [16] proposed a separate source-channel coding (SSCC) design using the maximal coding rate reduction (MCR2) principle to train the source encoder and optimize the precoding matrices. This approach maximizes separation between different classes of encoded representations in feature space, enabling Maximum A Posteriori (MAP) classification at the receiver. Although it addresses channel adaptation and improves model reusability, their method is restricted to the classification task.

Building on [16] that established clustered semantic representations in feature space for non-deep-learning-based channel coding design, we extend this concept; if we can understand how semantic representations influence task performance through their intrinsic properties, we could establish task-communication connections free from deep learning constraints. To realize this vision, we require both highly interpretable DNN models and reliable AI interpretability techniques. The Variational Autoencoder (VAE) [17], which introduced a probabilistic latent space assuming standard normal distribution of latent variables, achieves high interpretability and structured control in generative models. Its latent variables can be disentangled into independent semantic features, enabling the generation of smoothly interpolated samples through continuous interpolation. In AI interpretability research, attribution mechanisms aim to assign relevance scores (importance metrics) to features, quantifying their impact on model outputs. By strategically aligning these importance metrics with communication resource allocation, we can effectively bridge communication objectives with learning tasks.

Based on this rationale, we propose a novel SSCC framework in this paper, the semantic encoder and decoder exclusively handle source coding, while an attribution mechanism provides importance scores for semantic representations extracted by the semantic encoder. The channel coding scheme is then designed by integrating importance scores with communication scenarios. Under this architecture, no additional design is required for the semantic encoder’s output, and appropriate models for the semantic encoder and decoder can be flexibly chosen depending on task requirements. This approach enables adaptation to various downstream task types while improving model reusability.

In current research on importance-aware communication, the definition and measurement of importance depend on specific task scenarios. Existing studies employ diverse methods to measure importance for optimizing communication processes and ensuring task performance. For example, Weng et al. [14] directly linked semantic importance to text recovery accuracy in speech-to-text transmission tasks, enhancing semantic fidelity by precisely protecting critical information. Wang et al. [18] evaluated the impact weights of encoder neuron activation values through backpropagation of prediction errors in semantic prediction tasks to reduce waste of communication resources. At the data level, a data importance indicator [19] was proposed to dynamically quantify the effective value of samples in wireless channel environments by integrating the received SNR with data uncertainty. Another study [20] addressed communication resource constraints in edge machine learning scenarios by designing importance discrimination mechanisms under centralized frameworks (prioritizing misclassified data) and distributed frameworks (emphasizing local model data trained on large-scale datasets). Furthermore, Guo et al. [21] utilized pre-trained language modelto generate word importance rankings and implemented differentiated transmission protection, while Liu et al. [22] defined semantic importance by analyzing the correlation between semantic features and task performance, as well as inter-feature relationships, guiding resource allocation in digital semantic communications. These studies employ different approaches to define and utilize importance, yet all adopt task-oriented importance evaluation to optimize resource allocation, thus enhancing the efficiency and performance of communication systems. Although existing research covers various communication scenarios, the problem of importance-aware resource allocation in MIMO semantic communication has not been fully explored.

In our work, we investigate importance-aware resource allocation for MIMO semantic communication within the SSCC framework. We quantify importance based on each feature’s influence on the deep learning model’s inference performance, leveraging attribution mechanisms to derive importance scores for efficient resource allocation. We propose a communication-aware attribution method applicable to the VAE model, whose results can directly reflect the relative communication resource requirements for each feature, providing explicit guidance for resource allocation.

Our main contributions can be summarized as follows:

1. We propose an SSCC framework that addresses critical challenges in JSCC designs: parameter reusability limitations and adaptive channel condition handling. This paradigm enables flexible model deployment across diverse scenarios by decoupling semantic processing from channel optimization.
2. We propose a communication-inspired attribution method for VAE models by injecting controlled noise into semantic representations. Grounded in information bottleneck theory, our approach minimizes retained information while preserving task-critical features and perturbing non-essential ones. To characterize the importance score, we model the noise injection process as a communication channel, thereby endowing the derived importance scores with physical interpretability in communication contexts. Specifically, we establish a theoretical link between feature importance scores and channel capacity. This connection facilitates an importance-aware resource allocation strategy, bridging communication principles with deep learning objectives.
3. Systematic experiments validate both the effectiveness of the importance scores generated by our attribution method and their pivotal role in bridging learning tasks and communication optimization. Comparisons of resource allocation schemes demonstrate that our importance-aware approach achieves superior performance in learning tasks, highlighting the advantages of integrating importance awareness into communication resource management. This work provides valuable insights for SSCC semantic communication system design while expanding the applicability of explainable AI techniques in communication scenarios.

## 2. System Model

**Figure 1:**
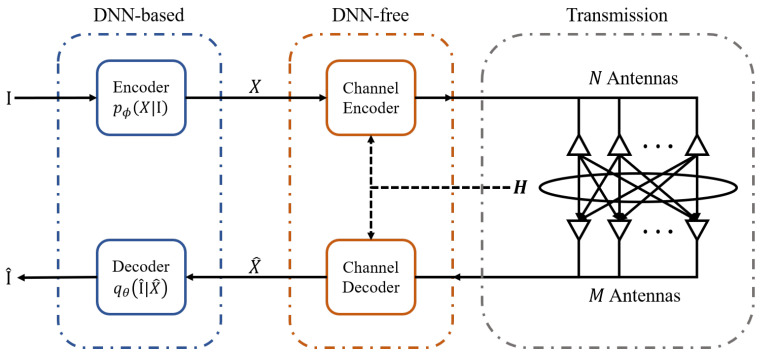
The framework of SSCC MIMO image transmission system.

As shown in Figure 1, we consider an SSCC MIMO image transmission system with *N* transmit antennas and *M* receive antennas. At the transmitter side, the DNN-based semantic encoder encodes the source image I into a low-dimensional semantic representation $X\in R^{m\times n}$. Distinct from JSCC designs, in our system, channel coding is decoupled from the semantic encoder and is not implemented via deep neural networks. After the DNN-free channel coding processing, the obtained symbols suitable for transmission are sent through the channel. At the receiver side, channel decoding is performed to obtain the estimated original semantic representation $\hat{X}\in R^{m\times n}$, followed by source decoding to reconstruct the transmitted image $\hat{I}$, completing the entire image transmission process.

### 2.1. Semantic Encoder and Decoder

In this paper, we adopt VAE as our semantic encoder and decoder. VAE is a generative framework based on probabilistic graphical models, learning latent space representations through variational inference. At the transmitter side, the input image I is encoded into a low-dimensional semantic representation *X* using a probabilistic encoder $p_{\phi }\left(X|I\right)$ parameterized by adjustable parameters. After channel transmission and signal recovery processes, $\hat{X}$ is obtained. The receiver employs the inference model $q_{\theta }\left(\hat{I}|\hat{X}\right)$ to reconstruct the image $\hat{I}$.

VAE assumes that latent variables $X\in R^{m\times n}$ follow a centered isotropic multivariate Gaussian prior, with the parametric encoder modeled as $p_{\phi }\left(X|I\right)=N\left(\mu_{X},\sigma_{X}^{2}i\right)$. To enable differentiable sampling, VAE introduces a reparameterization trick:

(1) $$\begin{array}{c} X=\mu_{\phi }\left(I\right)+\sigma_{\phi }\left(I\right)⊙\epsilon , \end{array}$$

where $\mu_{\phi }\left(I\right)\in R^{m\times n}$ and $\sigma_{\phi }\left(I\right)\in R^{m\times n}$ are the outputs of the encoder, ⊙ denotes the Hadamard product, $\epsilon \in R^{m\times n}$, and each element $\epsilon_{ij}$ is independently drawn from a standard normal distribution N(0,1). This operation shifts stochasticity to the input noise ϵ, allowing gradients to propagate to encoder parameters ϕ.

### 2.2. Channel Coding

**Figure 2:**
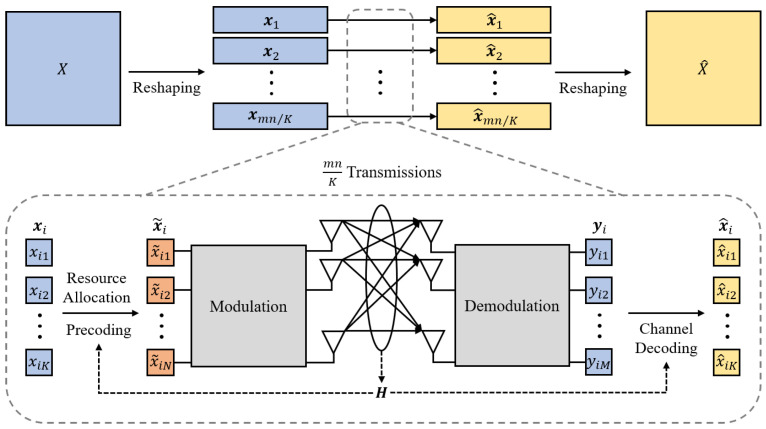
Channel coding process: the semantic representation is transformed into transmission-suitable symbols.

Following the extraction of semantic representations, the transmitter maps these representations to channel inputs. This mapping mechanism ensures reliable delivery of semantic information to the receiver even under channel uncertainty, a procedure we conceptualize as wide-sense channel coding. Specifically, the extracted semantic representation *X* is modeled as a continuous multivariate Gaussian signal, leading us to consider an analog communication system scenario. Unlike conventional channel coding in digital communication systems, the primary role of the channel coding module in this paper is to transform semantic representations into transmission-suitable symbols, while enhancing the robustness of the entire communication system via precoding, resource allocation, modulation, and demodulation. The process of channel coding is illustrated in Figure 2.

#### 2.2.1. Channel Encoder

Instead of developing new modulation components, we implemented the Amplitude Modulation (AM) scheme. In this paper, our research focuses on the design of precoding and resource allocation processes. Let the MIMO channel matrix be denoted as $H\in C^{M\times N}$, and let K=min(M,N), based on Singular Value Decomposition (SVD), H can be decomposed as:

(2) $$\begin{array}{c} H=U\Sigma V^{H}, \end{array}$$

where $U\in C^{M\times K}$ and $V\in C^{N\times K}$ are unitary matrices, and $\Sigma \in C^{K\times K}$ is a diagonal matrix with non-negative singular values arranged in descending order along its main diagonal. We denote Σ as $\mathrm{diag}(\sigma_{1},\ldots ,\sigma_{j},\ldots ,\sigma_{K})$, where $\sigma_{1}\geq \sigma_{2}\geq \cdots \geq \sigma_{K}$.

To adapt *X* for transmission and match the number of transmit antennas, *X* is reshaped into $x\in R^{K\times \frac{m\cdot n}{K}}$, which means that all available channels are utilized, with *K* symbols transmitted at a time, requiring $\frac{m\cdot n}{K}$ transmissions to complete sending *X* (or x). Let the *i*-th transmitted symbols be $x_{i}={\left[x_{i1},\ldots ,x_{ij},\ldots ,x_{iK}\right]}^{T}$.

Let $p_{i}$ denote the power allocation matrix for the *i*-th transmission, where $p_{i}\in R^{K\times K}$ is a diagonal matrix is defined as $\mathrm{diag}(\sqrt{p_{i1}},\ldots ,\sqrt{p_{ij}},\ldots ,\sqrt{p_{iK}})$, where $p_{ij}\left(j=1,2,\ldots ,K\right)$ denotes the power allocated to the *j*-th channel. To simplify the optimization conditions, we introduce the vector $\vec{x_{i}}=\left[x_{i1}^{2},\ldots ,x_{ij}^{2},\ldots ,x_{K}^{2}\right]$ for pre-processing $x_{i}$. After pre-processing and resource allocation, $x_{i}$ is transformed into transmission-ready symbols ${\tilde{x}}_{i}={\left[{\tilde{x}}_{i1},{\tilde{x}}_{i2},\ldots ,{\tilde{x}}_{iN}\right]}^{T}$, which is then distributed across the *N* antennas for transmission. The transformation from $x_{i}$ to ${\tilde{x}}_{i}$ can be expressed as:

(3) $$\begin{array}{c} {\tilde{x}}_{i}=Vp_{i}\frac{x_{i}}{\sqrt{∥\vec{x_{i}}{∥}_{2}}}. \end{array}$$

We assume that each transmission has the same total power limit $P_{\mathrm{total}}$, which requires:

(4) $$\begin{array}{c} E\left[∥{\tilde{x}}_{i}{∥}_{2}^{2}\right]=\mathrm{tr}\left({\tilde{x}}_{i}^{T}{\tilde{x}}_{i}\right)\leq P_{\mathrm{total}}. \end{array}$$

To simplify the power constraint, we introduce the vector $\vec{p_{i}}=\left[p_{i1},\ldots ,p_{ij},\ldots ,p_{iK}\right]$. Based on the Cauchy–Schwarz Inequality, we derive:

(5) $$\begin{array}{cc} \;E\left[∥{\tilde{x}}_{i}{∥}_{2}^{2}\right] & =\mathrm{tr}\left({\tilde{x}}_{i}^{H}{\tilde{x}}_{i}\right) \end{array}$$

(6) $$\begin{array}{cc} \; & =\mathrm{tr}\left({\left(Vp_{i}\frac{x_{i}}{\sqrt{∥\vec{x_{i}}{∥}_{2}}}\right)}^{H}Vp_{i}\frac{x_{i}}{\sqrt{∥\vec{x_{i}}{∥}_{2}}}\right) \end{array}$$

(7) $$\begin{array}{cc} \; & =\frac{1}{∥\vec{x_{i}}{∥}_{2}}\langle \vec{p_{i}},\vec{x_{i}}\rangle \end{array}$$

(8) $$\begin{array}{cc} \; & \leq ∥\vec{p_{i}}{∥}_{2}. \end{array}$$

This implies that by setting ${∥\vec{p_{i}}∥}_{2}=P_{\mathrm{total}}$, i.e., $\sum_{j=1}^{K}p_{ij}^{2}=P_{\mathrm{total}}^{2}$, we can satisfy the power constraint $E\left[∥{\tilde{x}}_{i}{∥}_{2}^{2}\right]\leq {∥\vec{p_{i}}∥}_{2}=P_{\mathrm{total}}$.

#### 2.2.2. Channel Decoder

At the receiver, the *M* antennas receive the symbols $y_{i}={\left[y_{i1},y_{i2},\ldots ,y_{iM}\right]}^{T}$ and $y_{i}=H{\tilde{x}}_{i}+w$. Following transmission of ${\tilde{x}}_{i}$, the received signal $y_{i}$ undergoes SVD post-processing with ${\tilde{y}}_{i}=U^{H}y_{i}$. The transmission process is derived as:

(9) $$\begin{array}{cc} \;{\tilde{y}}_{i} & =U^{H}y_{i} \end{array}$$

(10) $$\begin{array}{cc} \; & =U^{H}\left(U\Sigma V^{H}Vp_{i}\frac{x_{i}}{\sqrt{∥\vec{x_{i}}{∥}_{2}}}+w\right) \end{array}$$

(11) $$\begin{array}{cc} \; & =\Sigma p_{i}\frac{x_{i}}{\sqrt{∥\vec{x_{i}}{∥}_{2}}}+U^{H}w, \end{array}$$

where $w\in C^{M\times 1}$ is a noise vector indicating independent and identically distributed (i.i.d.) Gaussian noise following $w∼CN(0,\sigma_{w}^{2}I)$.

The recovered information ${\hat{x}}_{i}$ can be obtained from ${\tilde{y}}_{i}$ as:

(12) $$\begin{array}{cc} {\hat{x}}_{i} & =\sqrt{∥\vec{x_{i}}{∥}_{2}}p_{i}^{-1}\Sigma^{-1}{\tilde{y}}_{i} \end{array}$$

(13) $$\begin{array}{cc} \; & =\sqrt{∥\vec{x_{i}}{∥}_{2}}p_{i}^{-1}\Sigma^{-1}\left(\Sigma p_{i}\frac{x_{i}}{\sqrt{∥\vec{x_{i}}{∥}_{2}}}+U^{H}w\right) \end{array}$$

(14) $$\begin{array}{cc} \; & =x_{i}+\sqrt{∥\vec{x_{i}}{∥}_{2}}p_{i}^{-1}\Sigma^{-1}U^{H}w. \end{array}$$

After channel encoding and decoding processes are repeated $\frac{m\cdot n}{K}$ times until all $x_{i}$ are transmitted and the receiver obtains the complete recovered information $\hat{x}=\left[{\hat{x}}_{1},\ldots ,{\hat{x}}_{i},\ldots ,{\hat{x}}_{\frac{m\cdot n}{K}}\right]$. Then, $\hat{x}$ is reshaped into $\hat{X}$.

### 2.3. Optimization Objective

Since we consider an image transmission system as illustrated in Figure 1, the overall optimization objective of the system is to enhance the image reconstruction quality through communication optimization via power allocation. The optimization problem of the whole system can be formulated as follows:

(15) $$\min E\left[D\left(I,\hat{I}\right)\right]$$

(16) $$\;s.t.\;E\left[∥{\tilde{x}}_{i}{∥}_{2}^{2}\right]\leq P_{\mathrm{total}}$$

where D represents the distortion between the original image and the reconstructed image. We can measure this distortion in various ways, such as the Mean Squared Error (MSE) between the original and reconstructed images, the Peak Signal-to-Noise Ratio (PSNR) of the reconstructed image, or through downstream task evaluation.

Furthermore, by selecting different deep learning models to construct the semantic encoder and decoder, our proposed SSCC semantic communication system can be extended to various downstream tasks, such as classification, clustering, etc. Relevant experiments and discussions can be found in Section 4.4.

## 3. Methodology

The global optimization of resource allocation strategies constitutes a core challenge in achieving semantic communication efficiency maximization within SSCC system design. In the JSCC architecture, the system obtains parameter-fixed models through end-to-end joint training, whose performance ceiling is entirely determined by the neural network’s feature extraction and channel adaptation capabilities. In contrast, the SSCC architecture requires the semantic encoder to undergo an independent pre-training convergence process, with deployment in communication links only after stabilizing its basic task performance. Here, channel transmission quality governed by resource allocation strategies becomes the critical bottleneck constraining end-to-end semantic fidelity. This paradigm divergence leads SSCC systems to confront unique optimization propositions: given the established capability boundaries of semantic models, how to design adaptive communication resource allocation mechanisms that compensate for semantic distortion induced by channel distortion through establishing mapping relationships between communication conditions and learning task.

**Figure 3:**
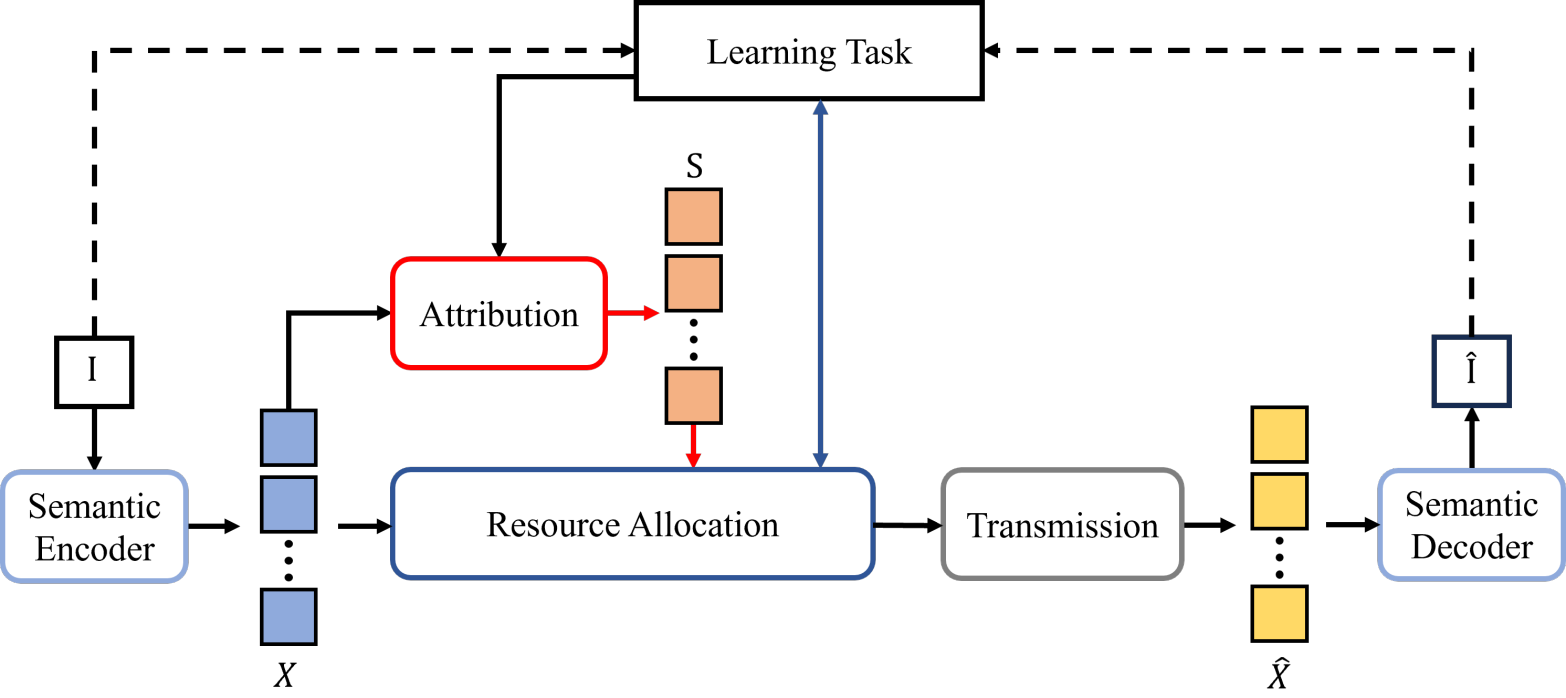
Illustration of importance-aware semantic communication framework.

To address this challenge, we propose an importance-aware semantic communication framework shown in Figure 3 that establishes connections between learning tasks and communication resource allocation. The image I is encoded into semantic representations *X* through a semantic encoder. An attribution mechanism analyzes the importance of each feature in *X* according to the learning task objective (15), generating importance scores S. The resource allocation strategy then combines these importance scores with the learning task objective to influence the feature transmission process, ensuring that the reconstructed image $\hat{I}$ recovered from $\hat{X}$ achieves the best possible quality, making the learning task objective as optimal as possible. We innovatively propose an attribution mechanism specifically designed for VAE models to establish connections between communication conditions and learning task performance. This framework establishes resource optimization equations with constrained conditions through quantitative evaluation of importance differences in semantic representations, providing SSCC system design with theoretical decision-making models that integrate both interpretability and adaptability.

### 3.1. Communication-Informed Bottleneck Attribution (CIBA)

In this paper, inspired by IBA [23], we propose a novel perspective to establish the connection between feature importance and communication. As discussed in Section 2.1, VAE assumes that latent variables $X\in R^{m\times n}$ follow a centered isotropic multivariate Gaussian prior, with the parametric encoder modeled as $p_{\phi }\left(X|I\right)=N\left(\mu_{X},\sigma_{X}^{2}I\right)$. Through the reparameterization trick, variables *X* can be viewed as a series of independent Gaussian signals. To estimate the importance of these Gaussian signals, we can add specific noise to each Gaussian signal to constrain the overall information flow, then feed the perturbed signals into the decoder to observe their impact on task performance.

**Figure 4:**
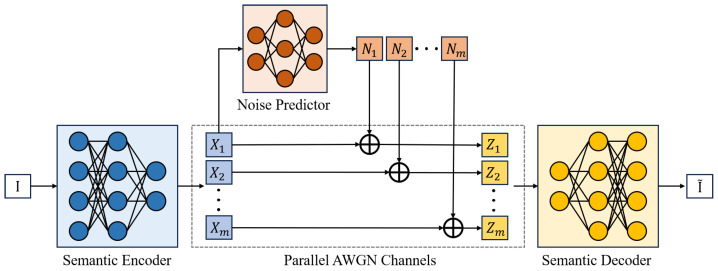
The framework of communication-informed bottleneck attribution method. For the convenience of presentation, we set n=1 and $X\in R^{m\times 1}$.

Figure 4 depicts the framework of the proposed CIBA method. We aim to find appropriate noise distributions that minimally affect the learning task, which implies that more important signals for the task will receive weaker noise (preserving information) while unimportant ones will be completely corrupted, thereby revealing the importance degree of each feature. Let $X\in R^{m\times n}$ be a feature matrix where each element $X_{ij}$ denotes the feature element at row *i* and column *j*. We assume that each $X_{ij}$ follows a normal distribution, i.e., $X_{ij}∼N\left(\mu_{X_{ij}},\sigma_{X_{ij}}^{2}\right)$. We can represent the entire matrix *X* using reparameterization. We have $X=\mu_{X}+\sigma_{X}⊙\epsilon$ and $X_{ij}=\mu_{X_{ij}}+\sigma_{X_{ij}}\epsilon_{ij}$, where ⊙ denotes the Hadamard product, $\epsilon \in R^{m\times n}$ and each element $\epsilon_{ij}$ is independently drawn from a standard normal distribution N(0,1).

Assuming independence between feature elements, we model the information flow constraint process as an analog additive white Gaussian noise (AWGN) channel communication process. In this process, m×n feature elements transmit through m×n parallel AWGN channels. For each element, the relationship is given by $Z_{ij}=X_{ij}+\Lambda_{ij}{\tilde{\epsilon }}_{ij}=X_{ij}+N_{ij},$ where ${\tilde{\epsilon }}_{ij}∼N\left(0,1\right)$ for all i=1,⋯,m and j=1,⋯,n, and $N_{ij}$ represents the analog additive white Gaussian noise at position (i,j).

The noise predictor, which is a neural network parameterized by α, adjusts $\Lambda_{ij}$ to control the power of the analog additive white Gaussian noise. We fix the parameters ϕ and θ of the semantic encoder and decoder, and only optimize the parameter α of the noise predictor through the information bottleneck objective.

(17) $$\begin{array}{c} \max\;I(I;Z)-\beta I(X;Z). \end{array}$$

This optimization objective aims to preserve more I-relevant information in *Z* while reducing the mutual information between *Z* and *X*. The hyperparameter β is used to control the relative importance of I(I;Z) and I(X;Z). For a small β, more bits of information are flowing and less for a higher value.

In this paper, we focus on image transmission, hence employing a VAE for the image reconstruction task, the reconstructed image denoted as $\tilde{I}$. $\tilde{I}$ is generated by the semantic decoder $q_{\theta }\left(\hat{I}|Z\right)$. We optimize I(I;Z) by minimizing the MSE between $\tilde{I}$ and I:

(18) $$\begin{array}{c} L_{1}\left(\alpha \right)=E_{\alpha }\left[{∥I-\tilde{I}∥}_{2}^{2}\right]. \end{array}$$

Considering $X_{ij}$ as a Gaussian signal, the AWGN channel capacity $C_{ij}$ becomes:

(19) $$\begin{array}{c} C_{ij}=\max I\left(X_{ij};Z_{ij}\right)=\frac{1}{2}\log\left(1+\frac{\mu_{X_{ij}}^{2}+\sigma_{X_{ij}}^{2}}{\Lambda_{ij}^{2}}\right). \end{array}$$

Under feature independence assumptions:

(20) $$\begin{array}{c} I\left(X;Z\right)=\sum_{i=1}^{m}\sum_{j=1}^{n}I\left(X_{ij};Z_{ij}\right)\leq \sum_{i=1}^{m}\sum_{j=1}^{n}\frac{1}{2}\log\left(1+\frac{\mu_{X_{ij}}^{2}+\sigma_{X_{ij}}^{2}}{\Lambda_{ij}^{2}}\right). \end{array}$$

Letting $L_{2}\left(\alpha \right)=E_{\alpha }\left[\sum_{i=1}^{m}\sum_{j=1}^{n}\frac{1}{2}\log\left(1+\frac{\mu_{X_{ij}}^{2}+\sigma_{X_{ij}}^{2}}{\Lambda_{ij}^{2}}\right)\right]$, we formulate the composite optimization objective:

(21) $$\begin{array}{c} L\left(\alpha \right)=L_{1}\left(\alpha \right)+\beta L_{2}\left(\alpha \right). \end{array}$$

The noise predictor model is optimized to minimize the loss function (21). We use stochastic gradient descent to train the model parameters to search for the optimal parameters $\alpha^{*}$ with a minimal L(α) as: $\alpha^{*}=\arg\min E\left[L(\alpha )\right]$, where the expectation is taken over the training datasets. It should be noted that during the training process, the parameters of the semantic encoder and decoder are fixed, and only the parameters of the noise predictor are updated. The loss function (21) is calculated through the original images, generated images, outputs of the semantic encoder, and outputs of the noise predictor.

This framework produces attribution results through the trained “noise power” predictor, specifically revealing the minimum channel capacity required to transmit each feature element $X_{ij}$ in the AWGN channel while minimally impacting learning task performance. Defining the importance score $S_{ij}=\frac{1}{2}\log\left(1+\frac{\mu_{X_{ij}}^{2}+\sigma_{X_{ij}}^{2}}{\Lambda_{ij}^{2}}\right)$, this metric indicates that higher importance features demand higher channel capacity. The importance score bridges learning task with communication objective, enabling rational communication resource planning that prioritizes high-importance features through enhanced resource allocation, thereby fully unleashing model potential.

### 3.2. Weighted Minimum Mean Squared Error (WMMSE)

As demonstrated in Section 3.1, each feature possesses distinct importance scores. To ensure alignment between communication objective and learning task, features with higher importance scores require prioritized fulfillment of their communication demands through enhanced resource allocation.

Minimum Mean Squared Error (MMSE) serves as a statistical estimation criterion aiming to minimize MSE between estimated and true values. Widely adopted in channel equalization, multi-user detection, and MIMO signal detection, it constitutes an indispensable tool in communication algorithm design. For MIMO communication scenarios, we formulate the power allocation optimization problem based on MMSE as follows:

(22) $$\begin{array}{cc} \min\; & E\left[{∥x_{i}-{\hat{x}}_{i}∥}_{2}^{2}\right] \end{array}$$

(23) $$\begin{array}{cc} s.t.\; & \sum_{j=1}^{K}p_{ij}^{2}=P_{\mathrm{total}}^{2}. \end{array}$$

Despite the widespread application of MMSE, its optimization objective is solely anchored to communication goals, which complicates its integration with learning tasks. We, therefore, enhance the MMSE scheme by incorporating importance scores into the optimization objective, proposing the WMMSE formulation:

(24) $$\begin{array}{cc} \;\min\; & \sum_{j=1}^{K}E\left[S_{ij}^{\gamma }{∥x_{ij}-{\hat{x}}_{ij}∥}_{2}^{2}\right] \end{array}$$

(25) $$\begin{array}{cc} s.t.\; & \sum_{j=1}^{K}p_{ij}^{2}=P_{\mathrm{total}}^{2}. \end{array}$$

The hyperparameter γ controls the magnitude of importance weights, with its value being empirically determined through systematic tuning.

Leveraging results from Equation (14), the optimization objective (24) simplifies to:

(26) $$\begin{array}{cc} \sum_{j=1}^{K}E\left[S_{ij}^{\gamma }{∥x_{ij}-{\hat{x}}_{ij}∥}_{2}^{2}\right] & =\sum_{j=1}^{K}\frac{S_{ij}^{\gamma }{∥\vec{x_{i}}∥}_{2}\sigma_{w}^{2}}{\sigma_{j}^{2}p_{ij}^{2}}. \end{array}$$

This leads to the refined optimization problem:

(27) $$\begin{array}{cc} \min\; & \sum_{j=1}^{K}\frac{S_{ij}^{\gamma }{∥\vec{x_{i}}∥}_{2}\sigma_{w}^{2}}{\sigma_{j}^{2}p_{ij}^{2}} \end{array}$$

(28) $$\begin{array}{cc} s.t.\; & \sum_{j=1}^{K}p_{ij}^{2}=P_{\mathrm{total}}^{2}. \end{array}$$

Introducing the Lagrange multiplier λ≥0, we construct the Lagrangian:

(29) $$\begin{array}{cc} L(p_{i1},\ldots ,p_{iK},\lambda ) & =\sum_{j=1}^{K}\frac{S_{ij}^{\gamma }{∥\vec{x_{i}}∥}_{2}\sigma_{w}^{2}}{\sigma_{j}^{2}p_{ij}^{2}}+\lambda \left(\sum_{j=1}^{K}p_{ij}^{2}-P_{\mathrm{total}}^{2}\right). \end{array}$$

Setting $\nabla_{p_{ij}}L\left(p_{i1},\ldots ,p_{iK},\lambda \right)=0$ yields optimal power allocation for the *j*-th subchannel during *i*-th transmission:

(30) $$\begin{array}{c} p_{ij}^{*}=\sqrt[{4}]{\frac{S_{ij}^{\gamma }{∥\vec{x_{i}}∥}_{2}\sigma_{w}^{2}}{\lambda^{*}\sigma_{j}^{2}}}. \end{array}$$

Substitution into the constraint gives:

(31) λ∗=∑j=1KSijγ∥xi→∥2σw2σj22Ptotal4.

The resultant transmission MSE per symbol becomes the following:

(32) $$\begin{array}{cc} E{\left[{∥x_{ij}-{\hat{x}}_{ij}∥}_{2}^{2}\right]}^{*} & =\sqrt{\frac{\lambda^{*}{∥\vec{x_{i}}∥}_{2}\sigma_{w}^{2}}{S_{ij}^{\gamma }\sigma_{j}^{2}}}. \end{array}$$

WMMSE explicitly incorporates importance scores $S_{ij}$, making MSE inversely proportional to both channel gain $\sigma_{j}$ and importance score $S_{ij}$. This dual dependency ensures that high-importance features receive superior communication conditions, effectively aligning communication objectives with learning tasks. Additionally, since the optimization objective (26) are inversely proportional to channel gains, we can introduce subchannel allocation to adjust the channel gain of each feature during transmission, thereby making the optimization objective (26) as small as possible. To achieve this goal, we adopt the following subchannel allocation scheme:

Sort features $x_{i}={\left[x_{i1},\ldots ,x_{iK}\right]}^{T}$ by descending importance scores $S_{ij}^{*}$ to obtain $x_{i}^{*}=\left[x_{i1}^{*},\ldots ,x_{iK}^{*}\right]$ with $S_{i1}^{*}\geq \cdots \geq S_{iK}^{*}$. Given SVD-derived subchannel gains $\sigma_{j}$ ordered as $\sigma_{1}\geq \cdots \geq \sigma_{K}$, we implement pairwise matching $\left(x_{ij}^{*},\sigma_{j}\right)$, establishing allocation configuration $\left\{\left(x_{i1}^{*},\sigma_{1}\right),\ldots ,\left(x_{iK}^{*},\sigma_{K}\right)\right\}$. This configuration minimizes the weighted optimization objective $\sum_{j=1}^{K}E{\left[S_{ij}^{\gamma }{∥x_{ij}^{*}-{\hat{x}}_{ij}^{*}∥}_{2}^{2}\right]}^{*}$.

The WMMSE framework achieves systematic trade-offs between communication efficiency and learning performance by prioritizing high-importance features through joint power allocation and subchannel matching, thereby fully unleashing model inference potential. Even if the optimal subchannel allocation is not adopted, the introduction of the importance score factor can slightly counterbalance the effects of channel gain.

In our proposed scheme, compared with the JSCC approach, the channel coding is decoupled and implemented through SVD precoding combined with solving the WMMSE optimization problem. This design ensures that under given channel state information conditions, the channel coding scheme yields a unique closed-form solution. Consequently, our proposed SSCC framework can adapt to channel variations while keeping model parameters fixed, thereby enhancing the reusability of DNN models and effectively addressing channel adaptation challenges.

## 4. Experiments and Discussion

In this section, we present a comparative analysis of the resource allocation schemes outlined in Section 3.2 and Appendix B within a 4×4 MIMO communication scenario. Specifically, each element of the channel matrix H is modeled to follow a complex normal distribution, denoted as H(i,j)∼CN(0,1). To quantify the signal quality, we define the average received SNR using the following formula:

(33) $$\begin{array}{cc} \mathrm{SNR} & =10\log_{10}\frac{E[∥H{\tilde{x}}_{i}{∥}_{F}^{2}]}{E[∥w{∥}_{F}^{2}]}\;\left(\mathrm{dB}\right). \end{array}$$

We conduct experiments to demonstrate the impact of semantic importance scores. We investigate the effect of parameter β on attribution performance, and explore the configuration of parameter γ in WMMSE. To evaluate the quality of the generated images, we adopt the PSNR metric. Simultaneously, we train an image classifier neural network termed the “evaluation model”, which classifies generated images to provide complementary quality assessment through classification accuracy. These experiments are conducted under various SNR scenarios using the MNIST dataset [24], which comprises 60,000 training images and 10,000 test grayscale images of handwritten digits (0–9). Detailed information about the experiments can be found in Appendix A.

### 4.1. Resource Allocation Schemes Comparison

In the Appendix B, we also introduce two baseline power allocation schemes named MMSE and Equal Power Allocation (EPA) that do not consider feature importance, along with their corresponding subchannel allocation strategies. Let WMMSE-M, MMSE-M, and EPA-M represent the proposed WMMSE, MMSE, and EPA with subchannel matching.

We experimentally test and obtain the Empirical Upper Bound (EUB) of the performance of the semantic encoder and decoder without communication processes. We use PSNR metrics and the evaluation model for comparative analysis of all six schemes.

**Figure 5:**
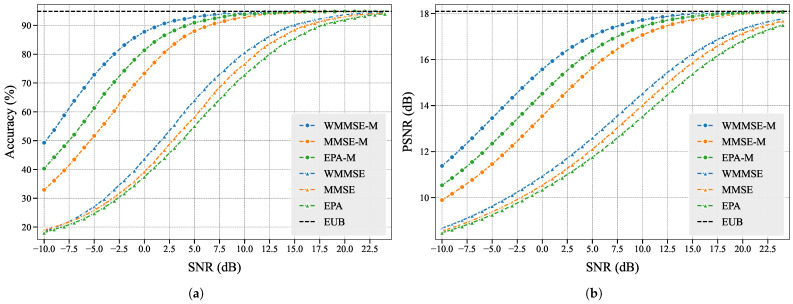
Performance comparison of resource allocation schemes: (**a**) Classification accuracy of evaluation model; (**b**) Reconstruction quality measured by PSNR.

As shown in Figure 5, we observe that WMMSE-M achieves superior performance in both evaluation dimensions, and compared to other schemes, it maximizes the potential of the semantic encoder and decoder across various SNRs, which aligns with our design principles. The outperformance of WMMSE over MMSE and EPA stems from its incorporation of semantic importance scores to bridge learning tasks and communication objectives, whereas MMSE and EPA completely neglect learning task considerations. The enhanced performance of WMMSE-M, MMSE-M, and EPA-M compared to their counterparts demonstrates the effectiveness of our subchannel matching scheme: by assigning higher-importance features to channels with stronger gains, we ensure more accurate transmission of critical features, thereby improving learning task performance.

Notably, while EPA-M outperforms MMSE-M, EPA underperforms MMSE. This phenomenon reflects the amplified impact of channel gain variations in EPA-based schemes, consistent with our theoretical analysis in Appendix B.2. These experimental results collectively validate that our proposed CIBA attribution method effectively evaluates importance levels of the features.

### 4.2. Semantic Importance Score Validation

In Section 3.1, we know the noise predictor provides the maximum tolerable noise power $\Lambda_{ij}$ for each feature $X_{ij}$, thus we can derive the importance score, denoted by $S_{ij}$, for each feature $X_{ij}$ as:

(34) $$\begin{array}{cc} S_{ij} & =\frac{1}{2}\log\left(1+\frac{\mu_{X_{ij}}^{2}+\sigma_{X_{ij}}^{2}}{\Lambda_{ij}^{2}}\right). \end{array}$$

**Figure 6:**
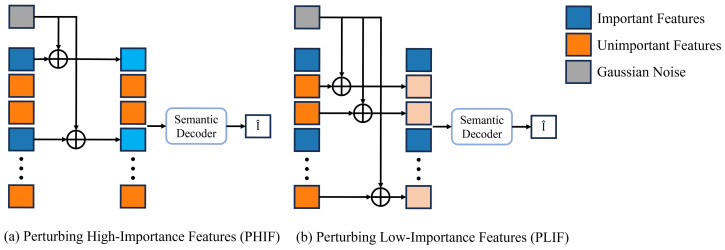
Illustration of two perturbation modes.

The physical meaning of the importance score $S_{ij}$ can be understood as the required channel capacity for the feature $X_{ij}$ in an AWGN channel. When the actual channel capacity of $X_{ij}$ during communication falls below $S_{ij}$, it will impact the performance of the deep learning task. To validate this perspective, after obtaining feature importance scores, we select a threshold $S_{T}$ and designate features with $S_{ij}\geq S_{T}$ as important features, while those with $S_{ij}<S_{T}$ are considered unimportant. Regarding the selection of $S_{T}$, we will provide further details later. We posit that important features are more critical for model inference and exhibit lower tolerance to Gaussian noise perturbations, whereas unimportant features show the opposite behavior. To verify this hypothesis, we propose two perturbation modes as shown in Figure 6: Perturbing High-Importance Features (PHIF) and Perturbing Low-Importance Features (PLIF). PHIF and PLIF, respectively, apply Gaussian noise perturbations to important and unimportant features. Under both perturbation modes, the perturbed features are fed into the semantic decoder to observe how changes in important versus unimportant features affect model inference.

**Figure 7:**
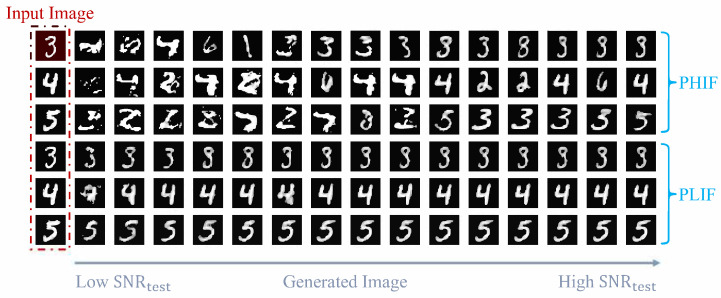
Comparison of PLIF and PHIF perturbations under varying ${\mathrm{SNR}}_{\mathrm{test}}$ conditions. PHIF perturbations induce more severe distortion than PLIF under increasing ${\mathrm{SNR}}_{\mathrm{test}}$.

We visually demonstrate the different impacts of high- and low-importance features by observing changes in generated images under these two perturbation modes. Figure 7 presents three groups of comparisons between PLIF and PHIF when $S_{T}$ is set to 3. The leftmost images in red boxes show the original input images, followed by generated results with progressively increasing Gaussian noise power (from left to right). We use the signal-to-noise ratio ${\mathrm{SNR}}_{\mathrm{test}}$ between features and added noise to represent perturbation intensity. The upper three rows show PHIF perturbations, while the lower three rows show PLIF perturbations. Increasing progressively ${\mathrm{SNR}}_{\mathrm{test}}$, we observe that distortion in important features causes significant changes in generated images, whereas distortion in unimportant features has minimal impact on generated images.

**Figure 8:**
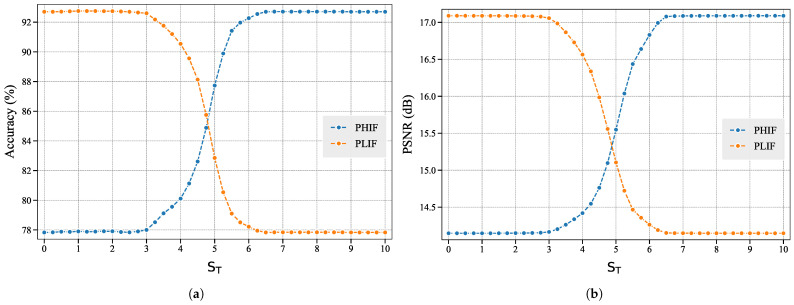
Effects of two perturbation modes on model performance at different $S_{T}$ (**a**) Classification accuracy of evaluation model; (**b**) Reconstruction quality measured by PSNR. The experimental results indicate that low-importance features have negligible impact on learning task performance, while high-importance features dominate model decision-making.

Subsequently, we investigate the impact of two perturbation modes on the model’s performance under the dynamic change of $S_{T}$. Across a range of different importance score thresholds $S_{T}$, PHIF and PLIF apply Gaussian noise perturbations with a fixed ${\mathrm{SNR}}_{\mathrm{test}}$ to important and unimportant features, respectively, resulting in distinct effects on model inference performance. The experimental results are shown in Figure 8. It can be observed that, under both perturbation modes, the model inference performance begins to change when $S_{T}\geq 3$. This suggests that features with importance scores $S_{ij}\geq 3$ are more critical for model inference and exhibit lower tolerance to noise, requiring better transmission conditions. Thus, $S_{T}=3$ can be a suitable threshold for separating important and unimportant features.

Furthermore, in the PHIF mode, where perturbations are applied to features satisfying $S_{ij}\geq S_{T}$, model performance gradually improves as $S_{T}$ increases because fewer high-importance features are perturbed. Similarly, in the PLIF mode, where perturbations target features with $S_{ij}<S_{T}$, model performance progressively deteriorates as $S_{T}$ increases, because more features of high importance are perturbed.

These experimental results demonstrate that low-importance features have negligible impacts on task performance, whereas high-importance features dominate model decision-making. High-importance features (which are fewer in quantity) preserve more critical information, exert greater influence on model decisions, and require better communication conditions for transmission. Therefore, incorporating considerations of feature importance is essential when designing resource allocation schemes in SSCC strategies.

### 4.3. Hyperparameter Selection

#### 4.3.1. Hyperparameter β Selection

The hyperparameter $\beta \in [10^{-3},10^{2}]$ is determined through grid search to identify the optimal β. We train our noise predictor under different β values with identical training epochs, and evaluate the performance of the EPA-M scheme using predicted importance scores from different predictors on the same task. Since EPA-M’s learning performance solely depends on subchannel allocation results where features are only affected by channel gains, this setup effectively reveals the connection between communication resources and feature importance.

**Figure 9:**
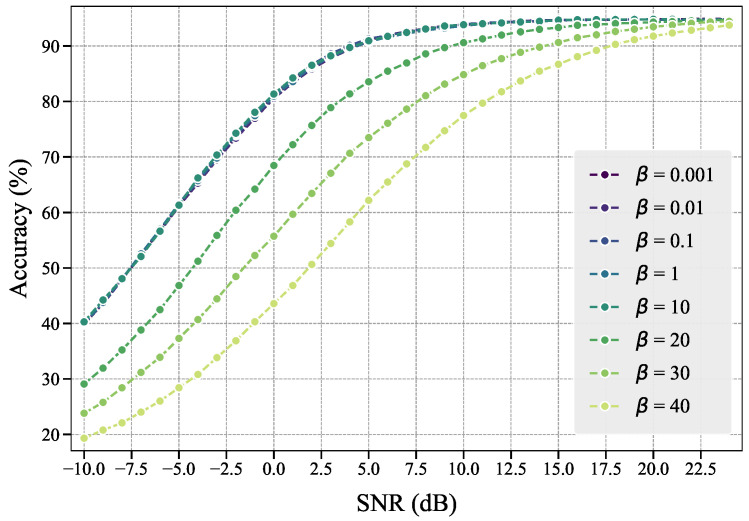
Performance variation in the EPA-M scheme under different β values. Stable performance is observed for $\beta \in [10^{-3},10]$.

Figure 9 demonstrates the performance variation in the EPA-M scheme across different β values. Superior EPA-M performance indicates more accurate importance score predictions by the noise predictor. Experimental results reveal that when $\beta \in [10^{-3},10]$, the EPA-M performance remains stable with satisfactory outcomes. However, as β increases beyond this range, excessive information flow constraints lead to significant dilution of task-relevant information, resulting in degraded prediction accuracy of the noise predictor and, consequently, diminished EPA-M performance. This empirical evidence suggests that setting $\beta \in [10^{-3},10]$ enhances the attribution effectiveness of the noise predictor.

#### 4.3.2. Hyperparameter γ Selection

**Figure 10:**
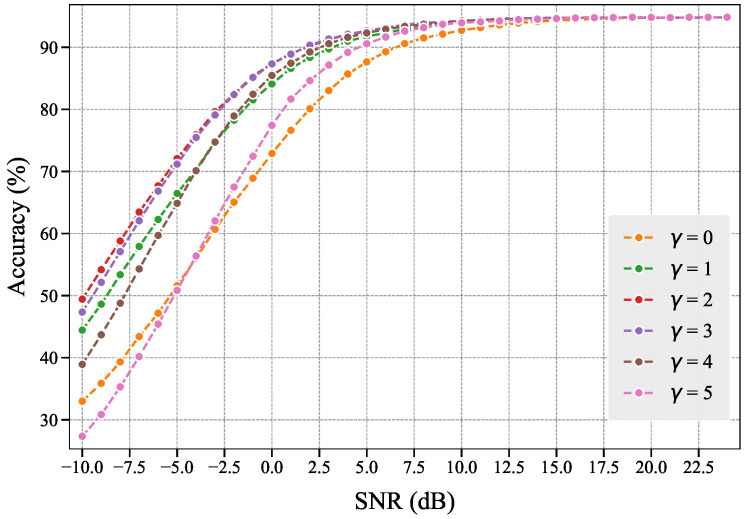
Performance variation in the WMMSE-M scheme under different γ values. Optimal performance occurs at γ=2.

Similarly, we determine the optimal hyperparameter γ∈[0,10] through grid search. We evaluate the performance of the WMMSE-M scheme under different γ values, with experimental results shown in Figure 10. Experimental results demonstrate that the WMMSE-M scheme achieves peak performance when γ approaches 2, while values below or beyond this range degrade scheme performance. When γ=0, the influence of $S_{ij}$ is eliminated, and WMMSE-M becomes equivalent to the MMSE-M scheme. When the value of γ is excessively large, due to limited allocatable communication resources, the highest-importance features may be allocated more communication resources than required, thereby preempting the resources intended for secondary-importance features. This ultimately leads to performance degradation of the entire communication system in learning tasks. Therefore, selecting an appropriate γ is crucial in importance-aware resource allocation schemes.

### 4.4. Extension to Classification Task

**Figure 11:**
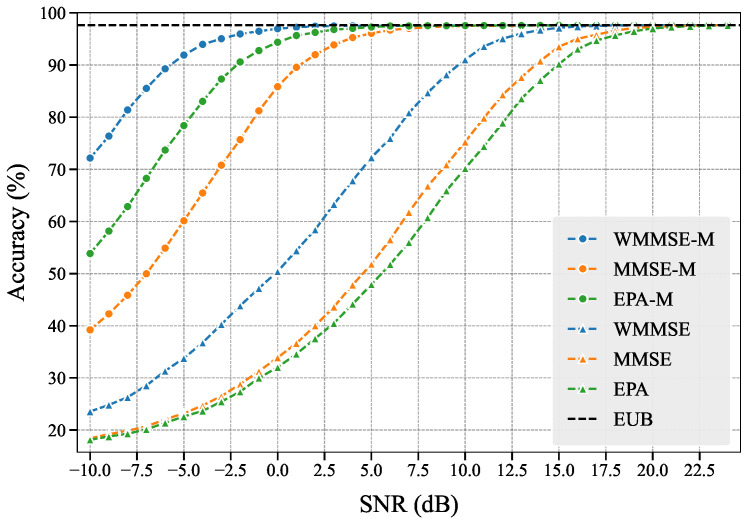
Performance comparison of resource allocation schemes for the image classification task.

The proposed SSCC semantic communication framework can construct semantic encoders and decoders using different deep learning models, thus the framework is not limited to image reconstruction task and can be extended to other applications. To validate this capability, we select a deep learning model for the image classification task to build the semantic encoder and decoder, with model parameters detailed in Table A2. The experimental setup is consistent with that in Section 4.1, and we use classification accuracy as the evaluation metric. Experimental results shown in Figure 11 demonstrate that our proposed WMMSE-M achieves the best performance, with schemes that incorporate subchannel matching outperforming those without. These findings confirm the applicability of our proposed method to image classification task, demonstrating that the proposed framework can be extended to classification task.

## 5. Conclusions

In this paper, we design and investigate an SSCC framework for semantic communication to address the pain points in JSCC design: low model reusability and channel adaptation issues. We propose a novel AI interpretability method called CIBA for VAE models, along with an importance-aware resource allocation strategy based on significance scores, to enable effective feature transmission in semantic communication systems and fully unleash the performance potential of semantic encoders and decoders. Through systematic experiments and analysis, we validate the effectiveness of the significance scores obtained by CIBA and their crucial role in bridging learning tasks and communication optimization. Comparative experimental results of two feature perturbation schemes (PHIF and PLIF) confirm the necessity of importance-aware resource allocation in semantic communication systems. By comprehensively comparing resource allocation schemes, we demonstrate that the importance-aware allocation scheme outperforms conventional approaches in learning task performance, highlighting the advantages of integrating importance awareness into communication resource management.

The proposed method opens up new possibilities for designing semantic communication systems, particularly in resource-constrained environments where intelligent resource allocation is crucial for maintaining semantic fidelity and task performance. Currently, our framework has only been validated on simple datasets, and future work will extend it to more complex datasets. While our CIBA interpretability scheme is currently specific to VAE models and simple communication scenarios (AWGN channels), future research should extend it to support more DNN architectures and sophisticated communication processes. Our importance-aware resource allocation scheme currently focuses on single-user MIMO power allocation and subchannel allocation, leaving the design of more complex resource allocation schemes for advanced communication scenarios as a valuable future work.

## Data Availability

Publicly available datasets were utilized in this study. The MNIST dataset can be accessed from Yann LeCun’s official website (http://yann.lecun.com/exdb/mnist/, accessed on 28 February 2025). Additionally, the dataset is available on Kaggle (https://www.kaggle.com/datasets/hojjatk/mnist-dataset, accessed on 28 February 2025).

## Appendix A. Experimental Details

### Appendix A.1. Model Training and Neural Network Architecture

We employ a VAE model to provide the semantic encoder and decoder required for the semantic communication process. The model parameters of the semantic encoder and decoder are listed in Table A1 and Table A2. The semantic encoder and decoder for image reconstruction task, trained on the MNIST dataset using the same training loss function in [17], handle image encoding and generation. The semantic encoder and decoder for classification task, trained on the MNIST dataset using cross-entropy loss, handle image encoding and classification. We adopt the PSNR metric to evaluate the quality of generated images. Simultaneously, we train an image classifier neural network termed the “evaluation model”, which classifies generated images to provide complementary quality assessment through classification accuracy. The parameters of the evaluation model are shown in Table A4. The evaluation model is trained on the MNIST dataset using cross-entropy loss.

After training the semantic encoder-decoder, we proceed to train the noise predictor. The parameters of the noise predictor are presented in Table A3. With the semantic encoder-decoder parameters fixed, the noise predictor takes the semantic encoder’s input as its input and produces outputs that feed into the semantic decoder. The loss function (21) is computed based on the decoder’s output, enabling gradient backpropagation to update the noise predictor’s parameters.

**Table A1:** The Neural Network Architecture for Semantic Encoder and Decoder (Image Reconstruction).

	Layer	Outputs
Encoder	Dense + ReLU	400
	Dense + ReLU	400
	[Dense →32 ($\mu_{X}$) / Dense →32 ($\sigma_{X}$)]	32 ($\mu_{X}+\sigma_{X}⊙\epsilon$)
Decoder	Dense + ReLU	400
	Dense + ReLU	400
	Dense + Sigmoid	28×28

**Table A2:** The Neural Network Architecture for Semantic Encoder and Decoder (Classification).

	Layer	Outputs
Encoder	Dense + ReLU	400
	Dense + ReLU	400
	[Dense →32 ($\mu_{X}$) / Dense →32 ($\sigma_{X}$)]	32 ($\mu_{X}+\sigma_{X}⊙\epsilon$)
Decoder	Dense + ReLU	400
	Dense + ReLU	400
	Dense + Softmax	10

**Table A3:** The Neural Network Architecture for Noise Predictor.

	Layer	Outputs
Predictor	Dense + ReLU	128
	Dense + ReLU	256
	Dense + ReLU	128
	Dense + ReLU	*32*

**Table A4:** The Neural Network Architecture of Evaluation Model for MNIST Classification Task.

	Layer	Outputs
Encoder	Flatten + Dense	400
Inference	Dense + ReLU	1024
	Dense + ReLU	256
	Dense + Softmax	10

### Appendix A.2. Evaluation Metrics

#### Appendix A.2.1. Peak Signal-to-Noise Ratio (PSNR)

The PSNR between a reference image I and a processed image $\hat{I}$ is defined as:

(A1) $$\mathrm{PSNR}=10\cdot \log_{10}\left(\frac{{\mathrm{MAX}}_{I}^{2}}{\mathrm{MSE}}\right)\;\left(\mathrm{dB}\right),$$

where:

- $\mathrm{MSE}=\frac{1}{HW}\sum_{h=1}^{H}\sum_{w=1}^{W}{\left[I\left(h,w\right)-\hat{I}\left(h,w\right)\right]}^{2}$
- H×W is the image resolution
- ${\mathrm{MAX}}_{I}$ denotes the maximum possible pixel value (typically 255 for 8-bit images)

#### Appendix A.2.2. Classification Accuracy

Classification accuracy is a fundamental metric for evaluating the performance of classification models, defined as the proportion of correctly predicted instances over the total number of samples. For the MNIST handwritten digit recognition benchmark, classification accuracy is computed as:

(A2) $$\mathrm{Accuracy}=\frac{1}{N_{\mathrm{test}}}\sum_{i=1}^{N_{\mathrm{test}}}I\left({\hat{y}}_{i}=y_{i}\right)$$

where:

- $N_{\mathrm{test}}$ is the standard test set size
- ${\hat{y}}_{i}$ denotes the predicted class label
- $y_{i}$ is the true label
- I(·) is the indicator function

This can be practically implemented using the confusion matrix for the 10-class problem:

(A3) $$\mathrm{Accuracy}=\frac{\sum_{k=0}^{9}C_{kk}}{\sum_{i=0}^{9}\sum_{j=0}^{9}C_{ij}},$$

where $C_{ij}$ represents the count of samples with true label *i* predicted as class *j*.

## Appendix B. Power Allocation Strategy

### Appendix B.1. Minimum Mean Squared Error (MMSE)

For MIMO communication scenarios, we formulate the power allocation optimization problem based on MMSE as follows:

(A4) $$\begin{array}{cc} \min\; & E\left[{∥x_{i}-{\hat{x}}_{i}∥}_{2}^{2}\right] \end{array}$$

(A5) $$\begin{array}{cc} s.t.\; & \sum_{j=1}^{K}p_{ij}^{2}=P_{\mathrm{total}}^{2}. \end{array}$$

Leveraging results from Equation (14), the optimization objective (A4) simplifies to:

(A6) $$\begin{array}{cc} E\left[{∥x_{i}-{\hat{x}}_{i}∥}_{2}^{2}\right] & =E\left[{∥\sqrt{∥\vec{x_{i}}{∥}_{2}}p_{i}^{-1}\Sigma^{-1}U^{H}w∥}_{2}^{2}\right] \end{array}$$

(A7) $$\begin{array}{cc} \; & =\sum_{j=1}^{K}\frac{{∥\vec{x_{i}}∥}_{2}\sigma_{w}^{2}}{\sigma_{j}^{2}p_{ij}^{2}}. \end{array}$$

This reduces to the optimization problem:

(A8) $$\begin{array}{cc} \min\; & \sum_{j=1}^{K}\frac{{∥\vec{x_{i}}∥}_{2}\sigma_{w}^{2}}{\sigma_{j}^{2}p_{ij}^{2}} \end{array}$$

(A9) $$\begin{array}{cc} s.t.\; & \sum_{j=1}^{K}p_{ij}^{2}=P_{\mathrm{total}}^{2}. \end{array}$$

Introducing Lagrange multiplier λ≥0, we construct the Lagrangian:

(A10) $$\begin{array}{cc} L(p_{i1},\ldots ,p_{iK},\lambda ) & =\sum_{j=1}^{K}\frac{{∥\vec{x_{i}}∥}_{2}\sigma_{w}^{2}}{\sigma_{j}^{2}p_{ij}^{2}}+\lambda \left(\sum_{j=1}^{K}p_{ij}^{2}-P_{\mathrm{total}}^{2}\right). \end{array}$$

Setting $\nabla_{p_{ij}}L\left(p_{i1},\ldots ,p_{iK},\lambda \right)=0$ yields optimal power allocation for the *j*-th subchannel during *i*-th transmission:

(A11) $$\begin{array}{c} p_{ij}^{*}=\sqrt[{4}]{\frac{{∥\vec{x_{i}}∥}_{2}\sigma_{w}^{2}}{\lambda^{*}\sigma_{j}^{2}}}. \end{array}$$

Substitution into the constraint gives:

(A12) λ∗=∑j=1K∥xi→∥2σw2σj22Ptotal4.

Under optimal power allocation, the transmission MSE per symbol becomes:

(A13) $$\begin{array}{cc} E{\left[{∥x_{ij}-{\hat{x}}_{ij}∥}_{2}^{2}\right]}^{*} & =\sqrt{\frac{\lambda^{*}{∥\vec{x_{i}}∥}_{2}\sigma_{w}^{2}}{\sigma_{j}^{2}}}. \end{array}$$

Notably, MMSE-optimized power allocation and transmission MSE depend solely on subchannel gain $\sigma_{j}$, with MSE inversely proportional to channel gain. This implies high-importance features assigned to low-gain subchannels receive fewer resources, potentially compromising task performance. To address this, we design subchannel allocation strategies prioritizing high-importance features through ordered matching:

Sort features $x_{i}={\left[x_{i1},\ldots ,x_{iK}\right]}^{T}$ by descending importance scores $S_{ij}^{*}$ to obtain $x_{i}^{*}=\left[x_{i1}^{*},\ldots ,x_{iK}^{*}\right]$ with $S_{i1}^{*}\geq \cdots \geq S_{iK}^{*}$. Given SVD-derived subchannel gains $\sigma_{j}$ ordered as $\sigma_{1}\geq \cdots \geq \sigma_{K}$, we implement pairwise matching $\left(x_{ij}^{*},\sigma_{j}\right)$, establishing allocation configuration $\left\{\left(x_{i1}^{*},\sigma_{1}\right),\ldots ,\left(x_{iK}^{*},\sigma_{K}\right)\right\}$. This strategy optimally aligns MMSE power allocation with learning task requirements.

### Appendix B.2. Equal Power Allocation (EPA)

We also design an equal power allocation scheme where equal power is allocated to all subchannels, satisfying $p_{i1}^{2}=\cdots =p_{ij}^{2}=\cdots =p_{iK}^{2}=\frac{P_{\mathrm{total}}^{2}}{K}$. The transmission MSE for each symbol $x_{ij}$ can be derived as:

(A14) $$\begin{array}{cc} E\left[{∥x_{ij}-{\hat{x}}_{ij}∥}_{2}^{2}\right] & =\frac{K{∥\vec{x_{i}}∥}_{2}\sigma_{w}^{2}}{P_{\mathrm{total}}^{2}\sigma_{j}^{2}}. \end{array}$$

It can be observed that under the EPA scheme, the transmission MSE for each symbol only relates to the channel gain and is inversely proportional to $\sigma_{j}^{2}$. Subchannels with higher channel gains provide better communication conditions. Similar to MMSE, to balance both learning and communication tasks, subchannel allocation is required to assign high-importance features to subchannels with higher channel gains. The allocation strategy is as follows:

Sort features $x_{i}$ by descending $S_{ij}^{*}$ to obtain $x_{i}^{*}$, then pair with ordered subchannels $\sigma_{j}$ through $\left\{\left(x_{i1}^{*},\sigma_{1}\right),\ldots ,\left(x_{iK}^{*},\sigma_{K}\right)\right\}$.

The transmission MSE of EPA scheme is inversely proportional to $\sigma_{j}^{2}$, while that of MMSE scheme is inversely proportional to $\sigma_{j}$. Therefore, under subchannel allocation strategy, the EPA scheme can provide smaller MSE for high-importance features, resulting in better learning task performance. Without this subchannel allocation strategy, if high-importance features are assigned to low-gain subchannels, EPA would introduce larger MSE for these features, leading to inferior learning performance compared to the MSE scheme.
